# Exogenous Application of Glycine Betaine Maintains Bioactive Compounds, Antioxidant Activity, and Physicochemical Attributes of Blood Orange Fruit During Prolonged Cold Storage

**DOI:** 10.3389/fnut.2022.873915

**Published:** 2022-06-24

**Authors:** Fariborz Habibi, Daniel Valero, María Serrano, Fabián Guillén

**Affiliations:** ^1^Department of Horticultural Science, School of Agriculture, Shiraz University, Shiraz, Iran; ^2^Department of Agro-Food Technology, University Miguel Hernández, Orihuela, Spain; ^3^Department of Applied Biology, University Miguel Hernández, Orihuela, Spain

**Keywords:** anthocyanin, citric acid, phenylalanine ammonia-lyase, sucrose, total phenols

## Abstract

Exogenous application of glycine betaine (GB) was evaluated on bioactive compounds, antioxidant activity, and physicochemical attributes of blood orange fruit cv. Moro at 3°C for 90 days. Vacuum infiltration (30 kPa) of GB was applied at 15 and 30 mM for 8 min. Parameters were measured after 1, 30, 60, and 90 days of storage plus 2 days at 20°C to simulate the shelf-life period. GB treatments significantly reduced weight and firmness losses in “Moro” blood orange fruit during cold storage. GB treatment maintained a higher concentration of organic acids (citric, malic, succinic, and oxalic acids) and sugars (sucrose, glucose, and fructose), especially for the higher GB doses (30 mM). During storage, GB treatments enhanced total anthocyanin concentration, total phenolic content, and total antioxidant activity. With respect to enzyme activities, the application of exogenous GB showed increases in catalase (CAT), ascorbate peroxidase, superoxide dismutase, phenylalanine ammonia-lyase, while suppressing the polyphenol oxidase activity. Overall, the most effective treatment was 30 mM GB leading to maintaining bioactive compounds, antioxidant activity, and quality in “Moro” blood orange fruit during long-term storage. The positive results would permit the use of GB as a postharvest tool to maintain the quality attributes of blood orange fruit.

## Graphical Abstract

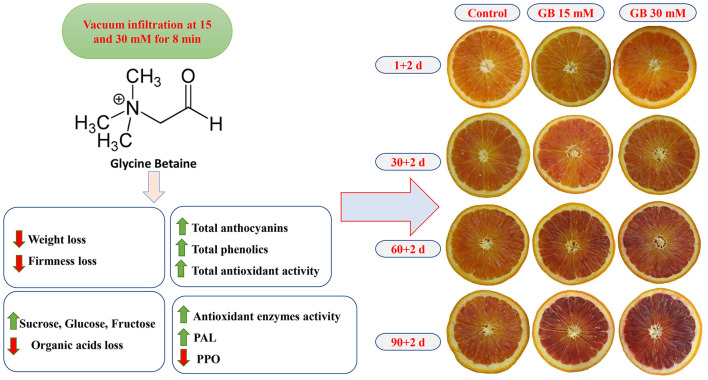

## Introduction

Blood oranges (*Citrus sinensis* L. Osbeck) fruit are a rich source of bioactive compounds compared to other citrus species since can promote the synthesis of anthocyanin pigments (1). Anthocyanin concentration can determine the internal quality of blood orange fruit and enhance antioxidant capacity, thus, very positive for human health by preventing some diseases (2).

Nowadays, postharvest treatment with elicitor compounds has been increased to preserve fruit quality during cold storage (1). For example, glycine betaine (GB) is a quaternary ammonium compound and can play a vital role in cell osmotic pressure, stabilization of protein and macromolecules, and enzyme function for enhancing chilling tolerance in higher plants (3, 4). Recently, postharvest treatment with GB has been done on horticultural crops, including sweet pepper (3), loquat (4), peach (5–7), zucchini (8), hawthorn (9) and pear (10–12), pomegranate (13), and banana (14).

Cold storage is the best strategy for increasing the postharvest life of blood oranges fruit for several months and provides the presence of this crop for market far from the harvest season (15). However, the susceptibility of blood oranges fruit to low temperatures can limit prolonged cold storage (16). In contrast, storage of blood oranges at ambient temperatures enhances weight loss, senescence, and fungal diseases, and the deterioration process subsequently decreases the shelf life (1). Cold temperature can reduce the physiological metabolism and senescence process, increase storability, and maintain the bioactive compounds of blood oranges fruit at long-term storage. The prolonged storage of blood oranges can induce quality losses, affecting fruit taste and some bioactive compounds (2). In this sense, there is a need for new techniques are crucial to increase storability and preserve the quality of blood orange fruit at cold storage.

In recent years, the postharvest application of elicitors has been applied for increasing storability and maintaining bioactive compounds of blood oranges fruit at prolonged cold storage (17). For the first time, the effect of postharvest treatment of GB was evaluated on maintaining the internal quality of blood orange fruit during cold storage. Therefore, the objective of this study was to assess the effect of postharvest GB treatment on bioactive compounds, antioxidant activity, and physicochemical attributes of “Moro” blood orange fruit during cold storage.

## Materials and Methods

### Fruit Treatments and Storage Conditions

Blood orange (*Citrus sinensis* L. Osbeck cv. Moro) fruit was picked up at the commercial maturity stage from a commercial citrus orchard in Jahrom, Fars province, Iran, and immediately transported to the postharvest laboratory. After harvest, the fruit was checked for homogeneity of size and no rind injuries and disinfected by dipping in a 2% sodium hypochlorite (NaOCl) solution. After drying the water, the fruits were treated with GB aqueous solution at 15 and 30 mM by vacuum infiltration at 30 kPa for 8 min. Control samples received no treatment. Treatments were achieved in three replicates of five fruit. All samples were stored for 90 days at 3°C and 90% relative humidity (RH). The storage duration and temperature were selected based on our previous work (18). The following parameters were evaluated after 1, 30, 60, and 90 days of cold storage plus 2 days at 20°C for shelf-life.

### Weight Loss and Firmness

Percentage of fruit weight loss (WL) was reported by weighing initial weight (IW) before storage and final weight (FW) for each sampling time (19).

Fruit firmness was measured with a texture analyzer (TA-XT2, UK) by compression of 10% of the equatorial area and reported as Newton (2).

### Chemical Attributes of Juice

Total soluble solids (TSS) and titratable acidity (TA) were determined with a refractometer and titration method, respectively, and TSS to TA ratio was reported by the division of them (20).

### Bioactive Compounds and Antioxidant Activity

Total anthocyanin concentration (TAC) was measured spectrophotometrically. Fruit juice was diluted (1:4) with potassium chloride (KCl) buffer (pH 1.0) and sodium acetate (C_2_H_3_NaO_2_) buffer (pH 4.5), and absorbance was read at 510 and 700 nm, and TAC reported as mg L^−1^ (20).

Total phenolic content (TPC) was measured using the Folin-Ciocalteu method (21). Briefly, 700 μl of juice was mixed with 900 μl of 2% sodium carbonate (Na_2_CO_3_), and then, 180 μl of 50% Folin was added after a 3 min reaction. Samples were read at 750 nm after 30 min keeping in a dark place at ambient temperature. TPC was calculated using a standard curve prepared from different concentrations of gallic acid and reported as mg gallic acid equivalents (GAE) L^−1^.

Total antioxidant activity (TAA) was measured with the 2,2-diphenyl-1-picrylhydrazyl (DPPH) method (22). Briefly, 100 μl of fruit juice was mixed with 1 ml DPPH (0.1 mM) and 1 ml Tris-HCl (pH = 7.5) buffer, and absorbance was read at 517 nm after 30 min keeping at ambient temperature and reported as a percentage.

### Individual Anthocyanin

Individual anthocyanins were quantified by high-performance liquid chromatography (HPLC) based on Martinez-Esplá et al. (23) method. For the preparation mixture, 0.5 g of lyophilized pulp was used as previously described (2). HPLC column and mobile phases (formic acid and acetonitrile) were as previously used. Major anthocyanins [cyanidin 3-glucoside and cyanidin 3-(6″-malonylglucoside)] were detected at 520 nm and quantified by standard curves and expressed as mg L^−1^.

### Individual Sugars and Organic Acids

Individual sugars and organic acids were determined by Martinez-Esplá et al. (23) method. For quantification, 0.5 g of lyophilized blood orange flesh was used, and mixture was prepared as previously described (2). Individual organic acids (citric acid, ascorbic acid, malic acid, oxalic acid, and succinic acid) and individual sugars (sucrose, glucose, and fructose) were expressed as g kg^−1^ using the standard curve.

### Assay of Enzymes Activity

Activities of enzymes were assayed spectrophotometrically in the flesh. First, 500 mg of fruit flesh was homogenized with extraction buffer (potassium phosphate, pH = 7). After centrifuge, the supernatant separated for catalase (CAT), peroxidase (POD), ascorbate peroxidase (APX), and superoxide dismutase (SOD) activities and total protein content. CAT and POD activities were evaluated by the Chance and Maehly (24) method. CAT activity was assessed based on hydrogen peroxide (H2O2) decomposition by the reduction of absorbance at 240 nm (24). POD activity was evaluated using guaiacol as substrate by reading the absorbance at 470 nm (24). APX activity was determined by measuring the absorbance at 290 nm as the amount of enzyme that oxidized ascorbate per minute (25). SOD activity was determined in a reaction mixture containing 100 μl of crude enzyme extract at 560 nm and calculated as the amount of enzyme that caused a 50% reduction of nitroblue tetrazolium (NBT) (26).

For PAL activity, briefly, 500 mg of fruit flesh was homogenized with sodium borate buffer (pH = 7). Then 0.5 ml crude enzyme extract, sodium borate buffer (pH = 8.8), and L-phenylalanine, and incubated at 37°C for 60 min. The PAL activity was measured at 290 nm (27).

For PPO enzyme extraction, 200 mg of fruit flesh was homogenized in potassium phosphate buffer (pH = 7.8). A reaction mixture, containing 100 μl of crude enzyme extract, potassium phosphate buffer (pH = 7), and pyrocatechol solution were incubated at 25°C for 10 min. Then, PPO enzyme activity was determined at 425 nm (28). The total protein content of enzyme extract was determined at 595 nm (29), and specific enzyme activities were reported as U mg^−1^ protein.

### Statistical Analysis

This study was planned as factorial based on a completely randomized design (CRD) with three replicates. Factors were treatment and sampling time. Data analysis, mean comparisons (LSD), and standard errors (SE) were performed with SAS (v. 9.4) software at *p* < 0.05.

## Results

### Weight Loss and Firmness

**Figure 1:**
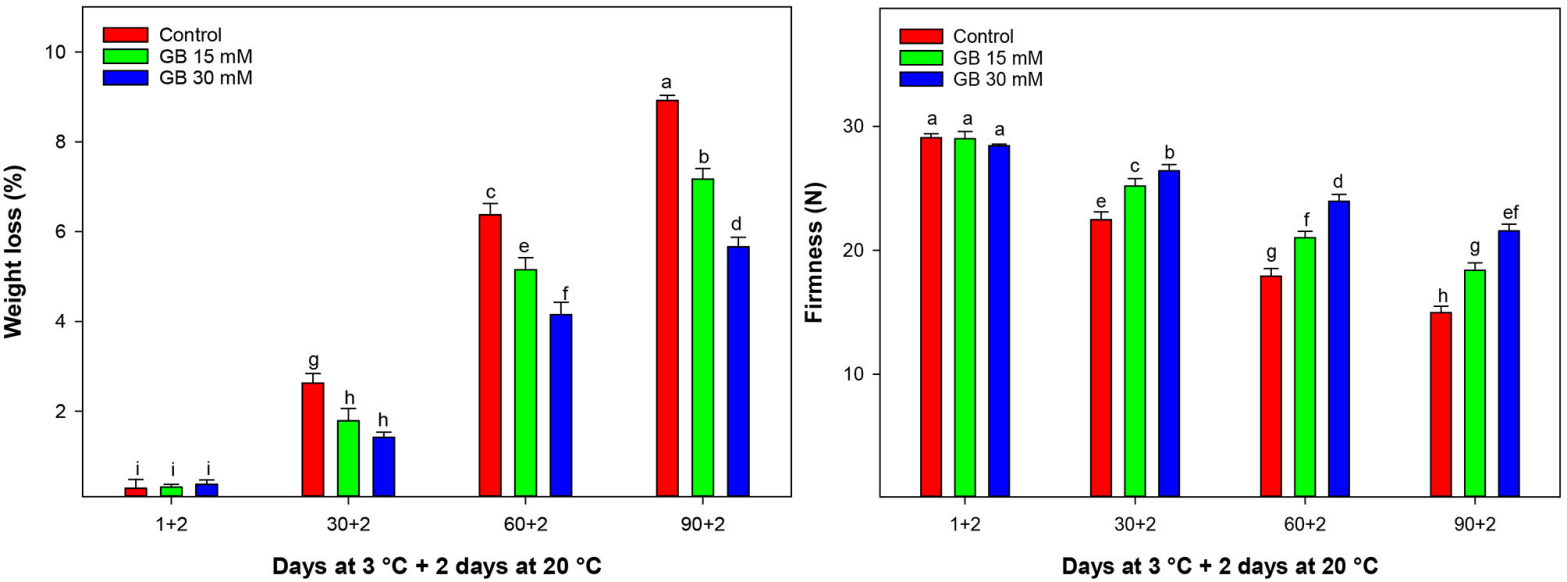
Changes in weight loss and fruit firmness in control and GB-treated fruit of “Moro” blood orange at 3°C. Different letters and vertical bars show a significant difference (*p* < 0.05) and standard errors (±SE) of means, respectively.

Weight loss (WL) increased during cold storage. In this sense, control fruit significantly had higher WL than GB-treated fruit (Figure 1). GB treatment alleviated WL, and the lowest WL was when 30 mM GB was applied. On the contrary, WL in control fruit was significantly higher compared to GB-treated fruit. In fact, at the last sampling time, WL in GB-treated fruit at 15 and 30 mM was 19.64 and 36.55% lower than in control fruit, respectively.

Firmness loss increased during cold storage. GB treatment maintained significantly higher blood orange fruit firmness during storage of “Moro” blood orange (Figure 1). Overall, the 30 mM GB treatment was an effective dose for preserving fruit firmness during cold storage. At the end of storage, firmness in GB-treated fruit at 15 and 30 mM was 18.64 and 30.66% higher than in control samples.

### Chemical Attributes of Juice

**Figure 2:**
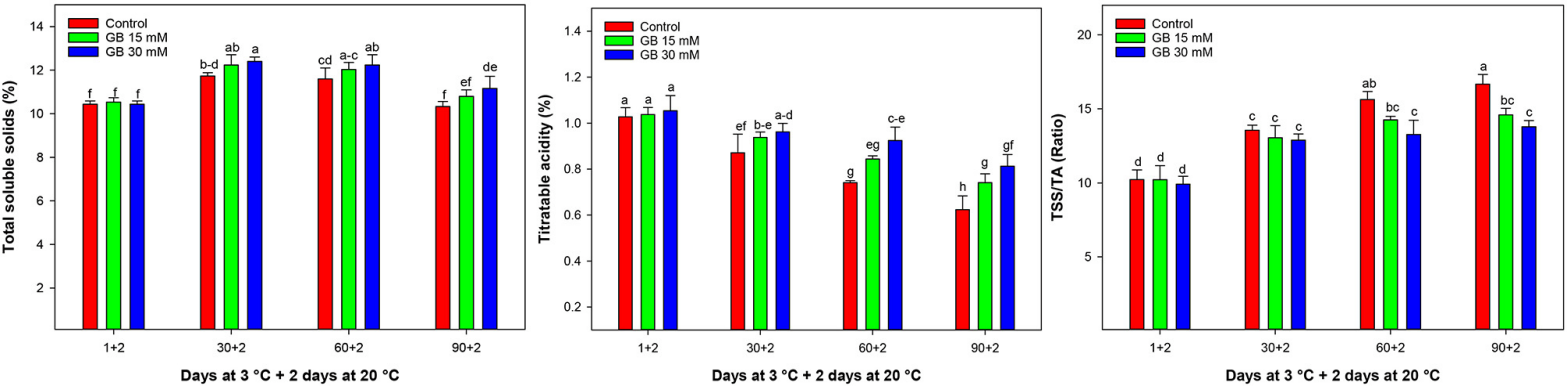
Changes in total soluble solids (TSS), titratable acidity (TA), and TSS/TA in control and GB-treated fruit of “Moro” blood orange at 3°C. Different letters and vertical bars show a significant difference (*p* < 0.05) and standard errors (±SE) of means, respectively.

TSS slightly increased in all samples up to 30 days and then remained constant or reduced during cold storage (Figure 2), with the highest and the lowest TSS being observed in 30 mM GB and control samples, respectively.

TA decreased in control and GB-treated during cold storage (Figure 2). However, control samples had the lowest TA at the end of cold storage. GB treatment reduced the TA losses, especially with the 30 mM concentration. After 90 days of cold storage, TA in fruit treated with 15 and 30 mM GB was 15.89 and 23.29% higher than in control fruit, respectively.

The ratio of TSS/TA was affected by all treatments applied during cold storage (Figure 2). GB-treated fruit had the lowest TSS/TA ratio at the last sampling time. In control samples, TSS/TA sharply increased during cold storage. Overall, 30 mM GB was the most effective dose for preserving TSS/TA ratio during cold storage.

### Individual Sugars and Organic Acids

**Figure 3:**
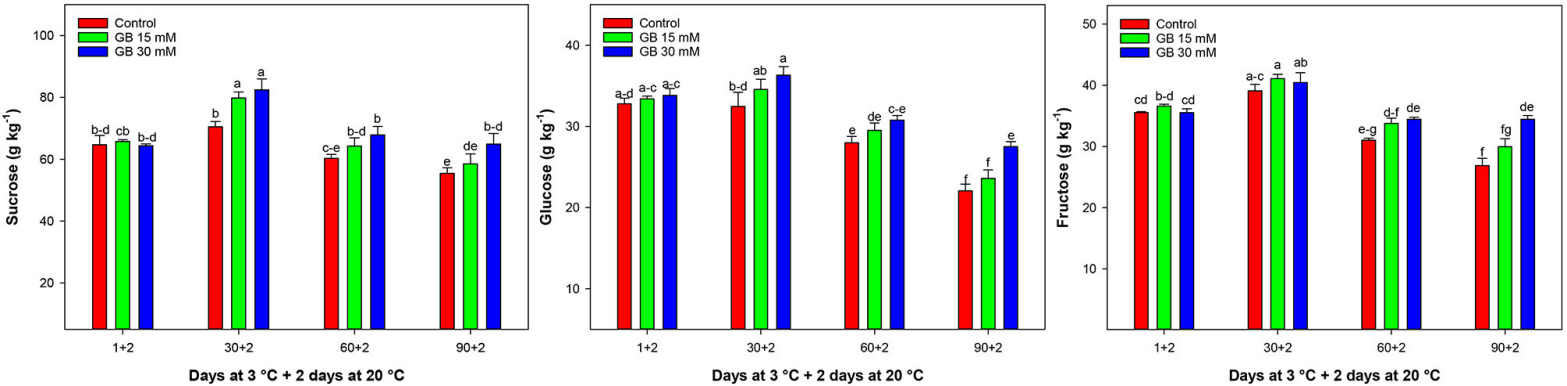
Changes in sucrose, glucose, and fructose in control and GB-treated fruit of “Moro” blood orange at 3°C. Different letters and vertical bars show a significant difference (*p* < 0.05) and standard errors (±SE) of means, respectively.

Individual sugars were affected by the postharvest GB treatment during storage (Figure 3), in which sucrose was found at a higher concentration than that glucose and fructose. Sucrose, glucose, and fructose increased up to 30 days in all treatments and then decreased to the end of cold storage. The reduction of individual sugars in GB-treated fruit was delayed compared to the control fruit after 90 days of cold storage. For example, the highest sucrose, glucose, and fructose levels found were in treated fruit with 30 mM GB.

**Figure 4:**
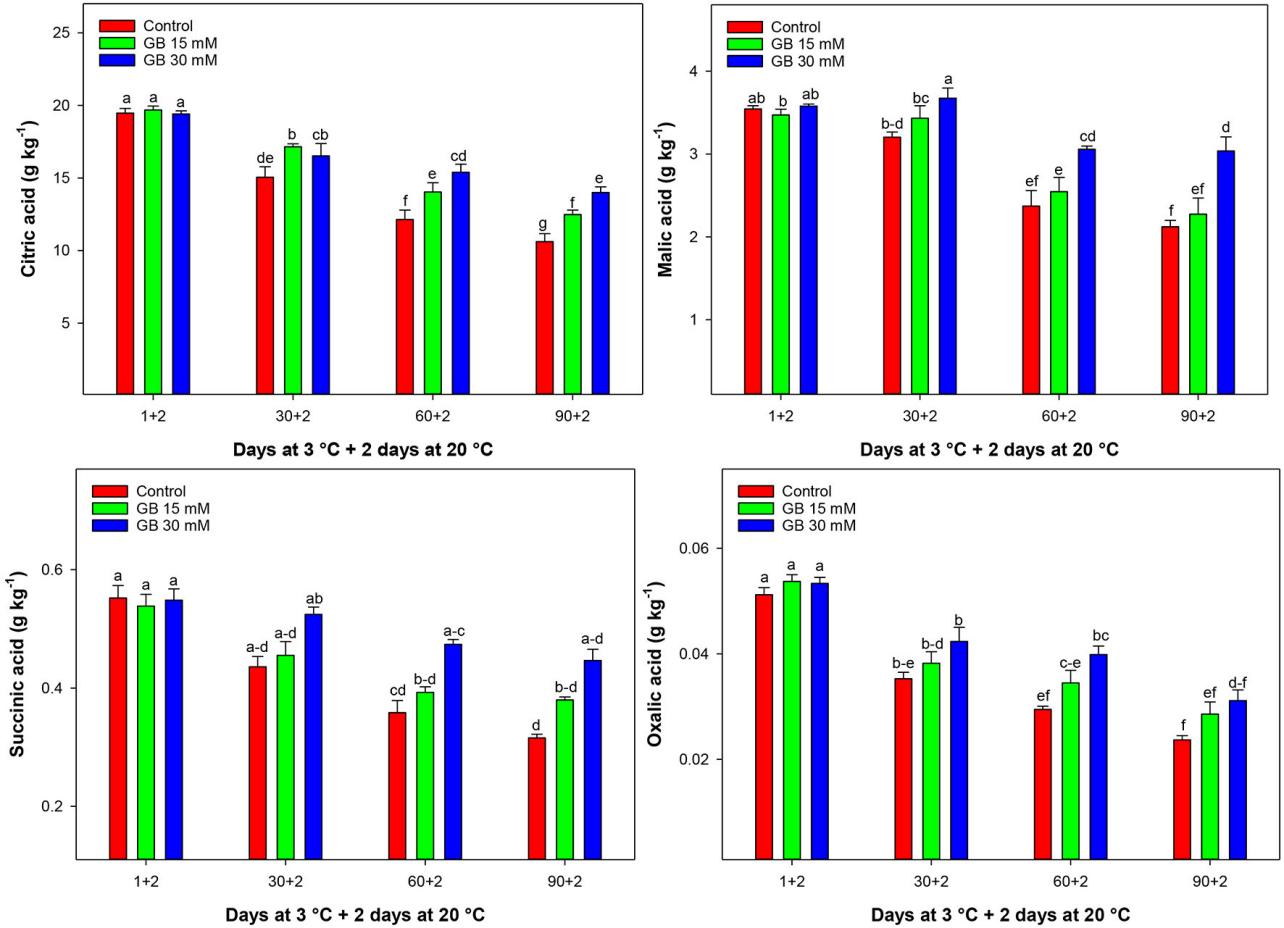
Changes in citric acid, malic acid, succinic acid, and oxalic acid in control and GB-treated fruit of “Moro” blood orange at 3°C. Different letters and vertical bars show a significant difference (*p* < 0.05) and standard errors (±SE) of means, respectively.

Citric, malic, succinic, and oxalic acids significantly decreased during cold storage for both control and treated fruit (Figure 4). GB-treated fruit had the highest content of citric, malic, succinic, and oxalic acids, especially for 30 mM GB during 90 days of storage, while sharply reduced in control samples. At the end of cold storage, the content of citric, malic, succinic, and oxalic acids in fruit treated with 30 mM GB, reached 24.2, 30.12, 29.33, and 23.93% more than in control samples, respectively.

### Bioactive Compounds and Antioxidant Activity

**Figure 5:**
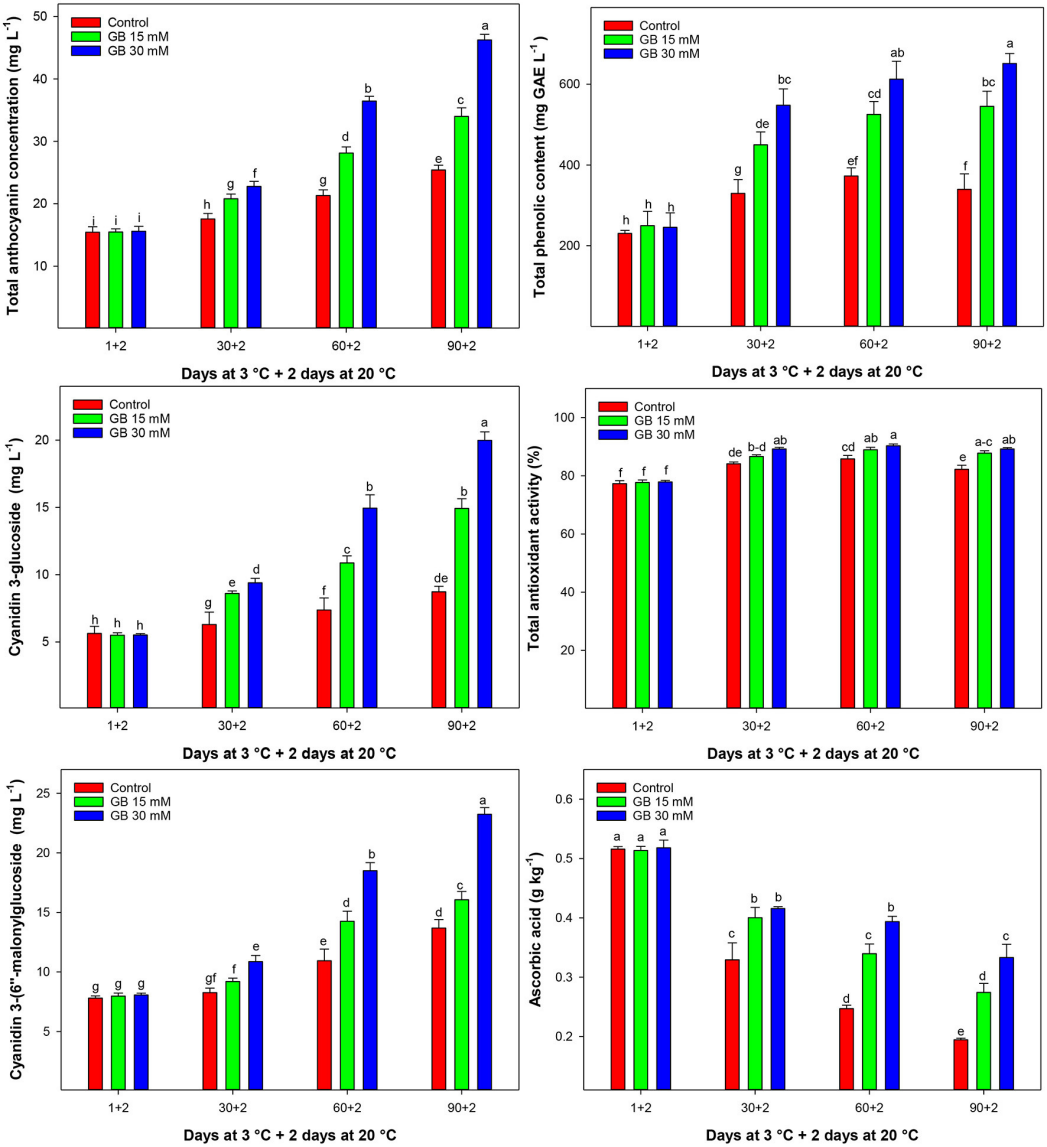
Changes in total anthocyanin concentration (TAC), cyanidin-3-glycoside, cyanidin 3-(6″-malonylglucoside), total phenolic content, total antioxidant activity (TAA), and ascorbic acid in control and GB-treated of “Moro” blood orange at 3°C. Different letters and vertical bars show significant difference (*p* < 0.05) and standard errors (±SE) of means, respectively.

Total anthocyanin concentration (TAC) increased during cold storage and was significantly enhanced by GB treatment (Figure 5). The highest content of TAC was observed at the end of storage in GB-treated fruit. At the last sampling time, TAC in fruit treated with 15 and 30 mM GB was 25.23 and 45.01% higher than in untreated fruit, respectively. Overall, the 30 mM GB was the most effective treatment for the enhancement of TAC during cold storage.

Major individual anthocyanins, including cyanidin 3-glucoside and cyanidin 3-(6″-malonylglucoside), were affected by treatments during cold storage (Figure 5). The trends of major individual anthocyanins were similar to TAC changes. Cyanidin 3-glucoside and cyanidin 3-(6″-malonylglucoside) contents were higher than in treated fruit with 30 mM GB during cold storage. At the end of cold storage cyanidin 3-glucoside and cyanidin 3-(6″-malonylglucoside) in fruit treated with 30 mM GB was 56.35 and 41.08% higher than in control samples, respectively.

Total phenolic content (TPC) increased up to 60 days of storage in all treatments and then remained constant in GB-treated or decreased in control samples (Figure 5). Control fruit showed the lowest TPC during cold storage. The 30 mM GB treatment enhanced TPC in blood orange fruit during cold storage. After 90 days of cold storage, TPC in treated fruit with 15 and 30 mM GB increased by 37.73 and 47.86% compared to control samples, respectively.

Total antioxidant activity (TAA) increased during cold storage and was affected by GB treatment (Figure 5). TAA increased up to 60 days of storage and then reduced to the end of storage. The highest and the lowest TAA were observed in treated fruit with 30 mM GB and control samples, respectively.

Ascorbic acid (AA) decreased during cold storage (Figure 5). Control fruit significantly had higher AA reduction than GB-treated fruit during cold storage. GB treatment delayed AA reduction in treated fruit, and the highest AA was found at 30 mM GB. At the end of storage, AA in GB-treated fruit at 15 and 30 mM was 29.07 and 41.59% higher than in control fruit, respectively.

### Antioxidant Enzymes Activity

POD activity was not detected in the flesh of fruit in all treatments for the whole sampling time.

**Figure 6:**
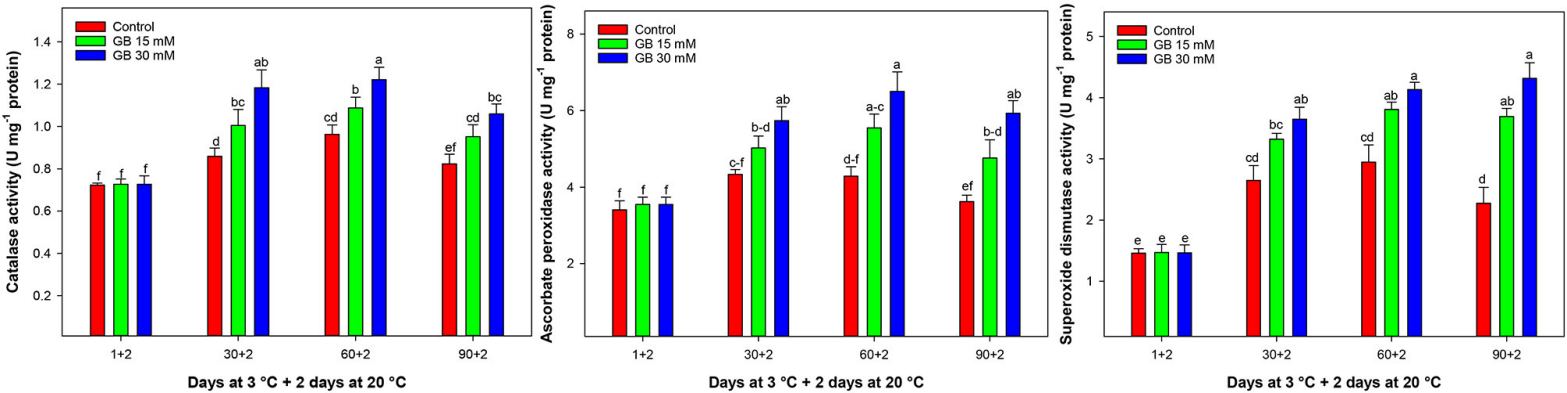
Changes in catalase (CAT), ascorbate peroxidase (APX), and superoxide dismutase (SOD) activities in control and GB-treated of “Moro” blood orange at 3°C. Different letters and vertical bars show significant difference (*p* < 0.05) and standard errors (±SE) of means, respectively.

CAT activity increased up to 60 days of cold storage and then decreased to the end experiment (Figure 6). Postharvest GB treatments significantly increased CAT activity in fruit. The lowest CAT activity was detected in the control samples. At the end of cold storage, the CAT activity in fruit treated with 15 and 30 mM GB was 13.53 and 23.32% higher than in control fruit, respectively.

APX activity was significantly enhanced by GB treatment compared to the control fruit during cold storage (Figure 6). APX activity increased up to 60 days of storage and then decreased in all treatments. The 30 mM GB was the most effective treatment for enhancing APX activity during cold storage.

SOD activity was also affected by GB treatments during storage (Figure 6). SOD activity increased up to 60 days of storage and then decreased in control fruit. At the 90 days of cold storage, the SOD activity in treated fruit with 15 and 30 mM GB was higher (38.39 and 47.28%, respectively) than in the control fruit.

### PAL and PPO Activities

**Figure 7:**
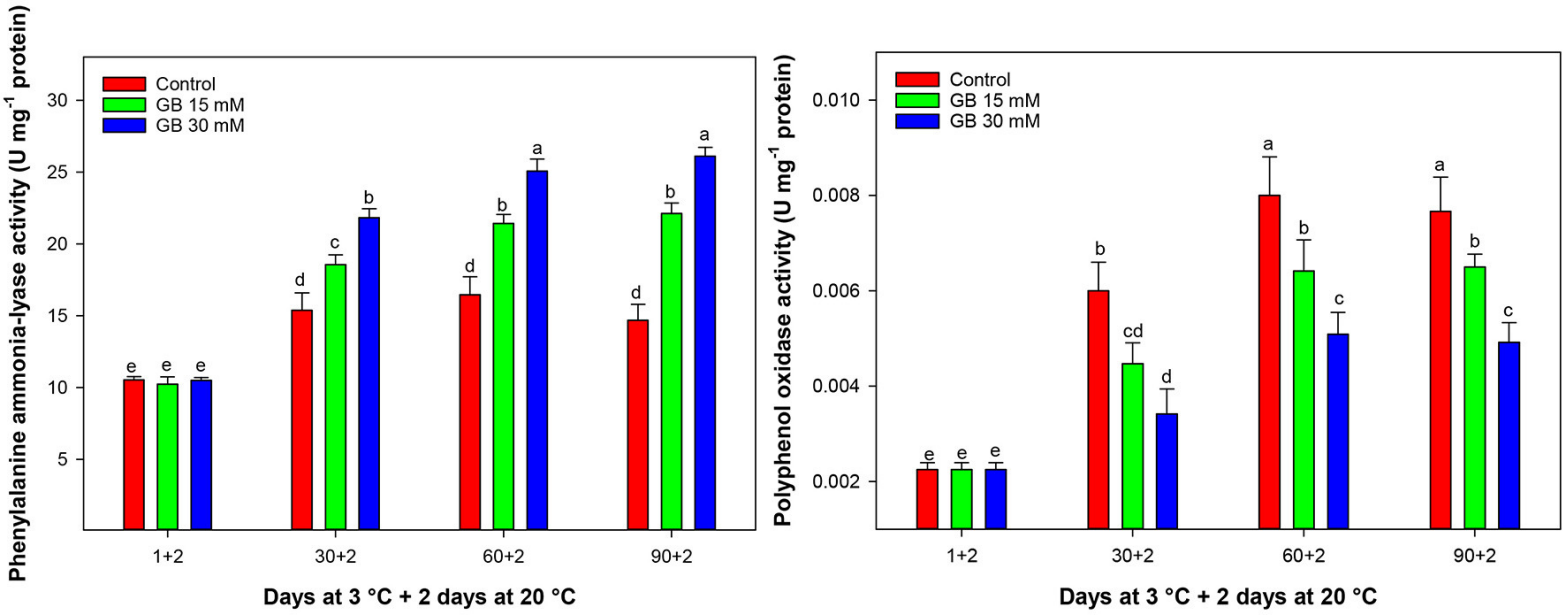
Changes in phenylalanine ammonia-lyase (PAL) and polyphenol oxidase (PPO) activities in control and GB-treated of “Moro” blood orange at 3°C. Different letters and vertical bars show significant difference (*p* < 0.05) and standard errors (±SE) of means, respectively.

PAL activity significantly increased by postharvest GB treatments during cold storage (Figure 7). PAL activity sharply increased in GB-treated fruit up to 60 days of storage and then remained constant. However, PAL activity decreased in control fruit after 60 days of cold storage. The highest and the lowest PAL activity was observed in 30 mM GB and control fruit, respectively. At the end of storage, the PAL activity in treated fruit with 15 and 30 mM GB was 33.59 and 43.75% greater than in control fruit, respectively.

PPO activity increased up to 60 days of cold storage and decreased constantly to the end of storage (Figure 7). The highest PPO activity was observed in the flavedo of control samples. GB treatment suppressed PPO activity during cold storage, and the lowest PPO activity was observed in the flavedo of treated fruit with 30 mM GB. After 90 days of storage, PPO activity in treated fruit with 15 and 30 mM GB was 15.21 and 35.86% lower than the control fruit, respectively.

## Discussion

Blood oranges consumption has gained enhanced acceptance and popularity among consumers due to its anthocyanin pigment content. Long-term cold storage increases the postharvest life of blood orange fruit and extends the presence of this commodity far from the harvest season for the market (30). However, long-term storage induces losses in organic acids, antioxidant activity, and bioactive compounds and subsequently affects fruit quality attributes of blood oranges. Thus, there is a challenge for producers and the fruit industry to solve this problem. Postharvest treatments with elicitors could be useful approaches for enhancing the storability of blood oranges at cold temperatures (17). In recent years, postharvest treatments with elicitors compounds maintained antioxidant activity and bioactive compounds of blood oranges fruit during cold storage (2, 17, 20). Numerous compounds, including polyamines (putrescine, Put) (20), γ-aminobutyric acid (GABA) (2), methyl jasmonate (MeJA) (2), methyl salicylate (MeSA) (2), and 24-epibrassinolide (24-EBR) (17), were applied after harvest on blood oranges. These compounds are classified as aliphatic polycations (Put), amino acids (GABA), and plant hormones (MeJA, MeSA, and 24-EBR). The application of elicitors was performed with vacuum infiltration (2, 20), vapor treatment (2), or dipping methods (17). The selection of treatment methods depends on compounds and facilities. However, the vapor treatment and dipping method are simple as compared to vacuum infiltration (30). In addition, the applied method depends on the nature of the substance. For example, it is possible using MeJA and MeSA by vapor treatment due to their volatile nature (2). In addition, some compounds can be applied using vacuum or dip in an aqueous solution. Although the dipping method is simple and fast, vacuum infiltration is more effective and provides optimal penetration of low doses into the fruit (20). In this study, we have applied postharvest treatment of GB for the first time to evaluate its effect on bioactive compounds, physicochemical attributes, antioxidant activity, and fruit quality of “Moro” blood orange during storage at 3°C.

GB treatment reduced WL in comparison to control samples during cold storage. Fruit WL is a result of water diffusion from the peel. Water is the main constituent of citrus fruit peel and maintaining peel moisture content can preserve fruit weight during cold storage. The moisture loss is mostly due to water diffusion through the peel surface and subsequently, let to fruit WL (19). On the other hand, WL is due to respiration, the transpiration process, and decreasing natural fruit wax during cold storage. Transpiration can be reduced by the natural fruit wax (31). In this study, 30 mM GB was effective for alleviating WL during cold storage, probably due to preserving membrane integrity at cold temperatures (18). Similarly, vacuum infiltration of GABA reduced WL in blood orange fruit. In this sense, vacuum infiltration is more effective than dipping methods (20). In addition, it seems that amino acids applied exogenously can maintain fruit moisture preserving peel epidermal structure (2).

The firmness of blood orange fruit is an important variable for market acceptability (19). In this study, GB-treated fruit had lower firmness loss than control fruit during cold storage. Pectic compounds are structural polysaccharides that maintain fruit firmness. The mechanism of firmness loss is due to pectin depolymerization by polygalacturonase, pectin lyase, pectin methylesterase, and cellulase enzymes that can induce some changes in cell wall composition and soften occur (31). In this study, GB treatment probably affected the activity of cell-wall degrading enzymes due to maintaining membrane stability and firmness. Vacuum infiltration of putrescine on blood orange cultivars to maintain firmness showed that treated fruit was firmer than the control samples due to binding to nucleic acids, phospholipids, and the carboxylic groups of polygalacturonic acid. Interestingly, this response was concentration-and cultivar-dependent (20).

Sucrose, glucose, and fructose are the main components of TSS (roughly 80%) of citrus fruit and other components are organic acids (10%), free amino acids, glucosides, proteins, and vitamins (2). In this study, GB-treated fruit had the highest TSS. The increase of TSS probably is due to the change of organic acids to sugars by the enzymatic process during cold storage (1).

In our study, the main individual sugars were sucrose, glucose, and fructose. All individual sugars increased up to 30 days and then reduced to the end of cold storage. The initial increase of individual sugars is due to conversion from organic acids. The reduction of individual sugars was probably because of the sugar consumption for energy sources supplied in the respiratory process during long-term storage (31). In addition, the decrease of sucrose content in blood oranges is a consequence of hydrolytic scission phenomena which mainly affect the sucrose and other glycosylated components of the juice during cold storage (17). However, GB treatment maintained a higher concentration of sucrose, glucose, and fructose and had a positive effect on preventing sugar reduction during cold storage. In pear fruit, GB treatment affected the sucrose, glucose, and fructose levels during cold storage, and control samples had more glucose, and fructose than GB-treated fruit (12).

Titratable acidity (TA) decreased during storage and GB-treated fruit had a higher TA at the end of storage. TA has been correlated with the content of organic acids and important components for quality citrus fruit juice (20). In this study, the main organic acids were citric, malic, ascorbic, succinic, and oxalic acids in the “Moro” blood orange fruit. Citric and malic acids were the first and second major organic acids respectively. Synthesis of sugars from organic acids, synthesis of phenolic compounds, and energy production are the main reasons for the reduction of organic acids during cold storage (1). In this study, fruit treated with 30 mM GB had higher organic acids during cold storage. The higher organic acids content in GB-treated fruit is probably due to an effect on respiration rate by reducing glycolytic enzyme activities, thus maintaining organic acids and delaying senescence increasing the fruit storability (32).

In this study, TSS/TA ratio increased during cold storage. Changes in organic acid content can affect this ratio. On the other hand, the acidity of citrus fruit is related to organic acid levels and organic acid loss due to conversion can change the TSS and TA ratio (33). In this study, fruit treated with 30 mM GB had a lower TSS/TA ratio during cold storage probably due to the higher organic acid content observed.

After harvesting, blood oranges fruit can synthesize anthocyanin by activating the enzymes involved in phenylpropanoid metabolism, mainly PAL enzyme under cold temperatures (34). In this study, PAL activity was enhanced in all treatments, and 30 mM GB was the most effective treatment for enhancing PAL activity. This enhancement probably is due to increasing ATP and ADP content which can be consumed for cellular metabolism (5). In addition, postharvest GB treatment increased PAL activity in hawthorn fruit enhancing anthocyanin accumulation (9). In addition, similar to TAC, cyanidin 3-glucoside and cyanidin 3-(6″-malonylglucoside) as major anthocyanins in blood oranges fruit were higher in GB-treated fruit. However, enhancement of TAC, cyanidin 3-glucoside, and cyanidin 3-(6″-malonylglucoside) in control samples was lower than in GB-treated fruit probably due to loss of organic acids or sugars, and subsequently lower ATP production for providing cellular energy at cold stress (1, 5). In addition, the degradation of anthocyanin can be influenced by PPO and POD activities (34). In this study, POD activity was not found in all treatments. In addition, PPO activity was higher than in comparison with GB-treated fruit. Therefore, PPO activity leads to anthocyanin molecule degradation. Higher PPO activity and lower PAL activity can explain why anthocyanin accumulation was lower in comparison with GB-treated fruit. However, PPO activity increased during storage probably due to increasing pH, reduction of organic acids, and fruit senescence and GB treatment delayed them (1). In this sense, GB-treated pomegranate fruit had more TAC than the control sample during cold storage (13). In addition, TAC and major anthocyanins increased in blood oranges with MeJA and MeSA more than with GABA treatments. MeJA and MeSA showed a higher effect when applied exogenously as compared to amino acids (GABA). It seems that plant hormones can obtain more beneficial effects than amino acids due to their nature and function on the fruit physiology after harvest (2). In this study, TPC increased in treated fruit and decreased in control samples at the end of storage. The increase of phenolic compounds is related to PAL activity and anthocyanin accumulation which led to an increment in the TPC (20). This is the main reason for higher TPC in GB-treated fruit. The reduction of TPC at the end of storage in control samples probably is due to enzymatic oxidation or degradation by POD and PPO enzyme activities (2). Concomitant with results, PAL activity increased by GB treatment and enhanced TPC in cold-stored pear fruit (12).

TAA increased and then reduced to the end of storage. Fruits under cold stress accumulate reactive oxygen species (ROS) and need to scavenge by enzymatic and non-enzymatic antioxidant systems (35). In this study, CAT, APX, and SOD increased up to 60 days of cold storage and then declined as a result of ROS overproduction. In addition, ascorbic acid and phenolic compounds can help with ROS scavenging as non-enzymatic antioxidants (31). In this study, ascorbic acid's decrease was probably due to the scavenging of ROS as an electron donor that can neutralize free radicals at suboptimal temperatures (2). In addition, ascorbate oxidase and POD activities can degrade ascorbic acid (1). In pear, APX, CAT, and SOD activities also increased rapidly in GB-treated fruit during cold storage (10).

## Conclusions

For the first time, we reported the effect of postharvest application of GB on bioactive compounds and antioxidant activity in blood orange fruit at prolonged cold storage. GB treatment delayed weight and firmness losses and organic acid and sugar accumulation. In addition, GB treatment enhanced bioactive compounds and maintained quality by increasing PAL and suppressing the PPO activity leading to a higher TAC and TPC, increasing TAA. It can be concluded that 30 mM GB was the most effective treatment and may be a promising strategy for preserving physicochemical, bioactive compounds, and antioxidant activity of blood orange fruit cv. Moro at 3°C.

## Data Availability Statement

The original contributions presented in the study are included in the article/Supplementary Material, further inquiries can be directed to the corresponding author.

## Author Contributions

FH and FG conceived and designed the experiment. FH performed the investigations, analytical determination and data analyses, and wrote the original draft. DV, MS, and FG edited the original draft. All authors contributed to the article and approved the submitted version.

## Funding

This manuscript belongs to the project Innovative and eco-friendly pre-and postharvest strategies with natural compounds to improve quality of fruits funded by Conselleria d'Innovació, Universitats, Ciència i Societat Digital (Generalitat Valenciana) through Prometeo Program (PROMETEO/2021/089).

## Conflict of Interest

The authors declare that the research was conducted in the absence of any commercial or financial relationships that could be construed as a potential conflict of interest.

## Publisher's Note

All claims expressed in this article are solely those of the authors and do not necessarily represent those of their affiliated organizations, or those of the publisher, the editors and the reviewers. Any product that may be evaluated in this article, or claim that may be made by its manufacturer, is not guaranteed or endorsed by the publisher.
